# Supplementary figures and images for: Macrophage- and CD4+ T cell-derived SIV differ in glycosylation, infectivity and neutralization sensitivity
Source: PLoS Pathog. 2024 May 28;20(5):e1012190. doi: 10.1371/journal.ppat.1012190 (PMC11161069; doi:10.1371/journal.ppat.1012190)

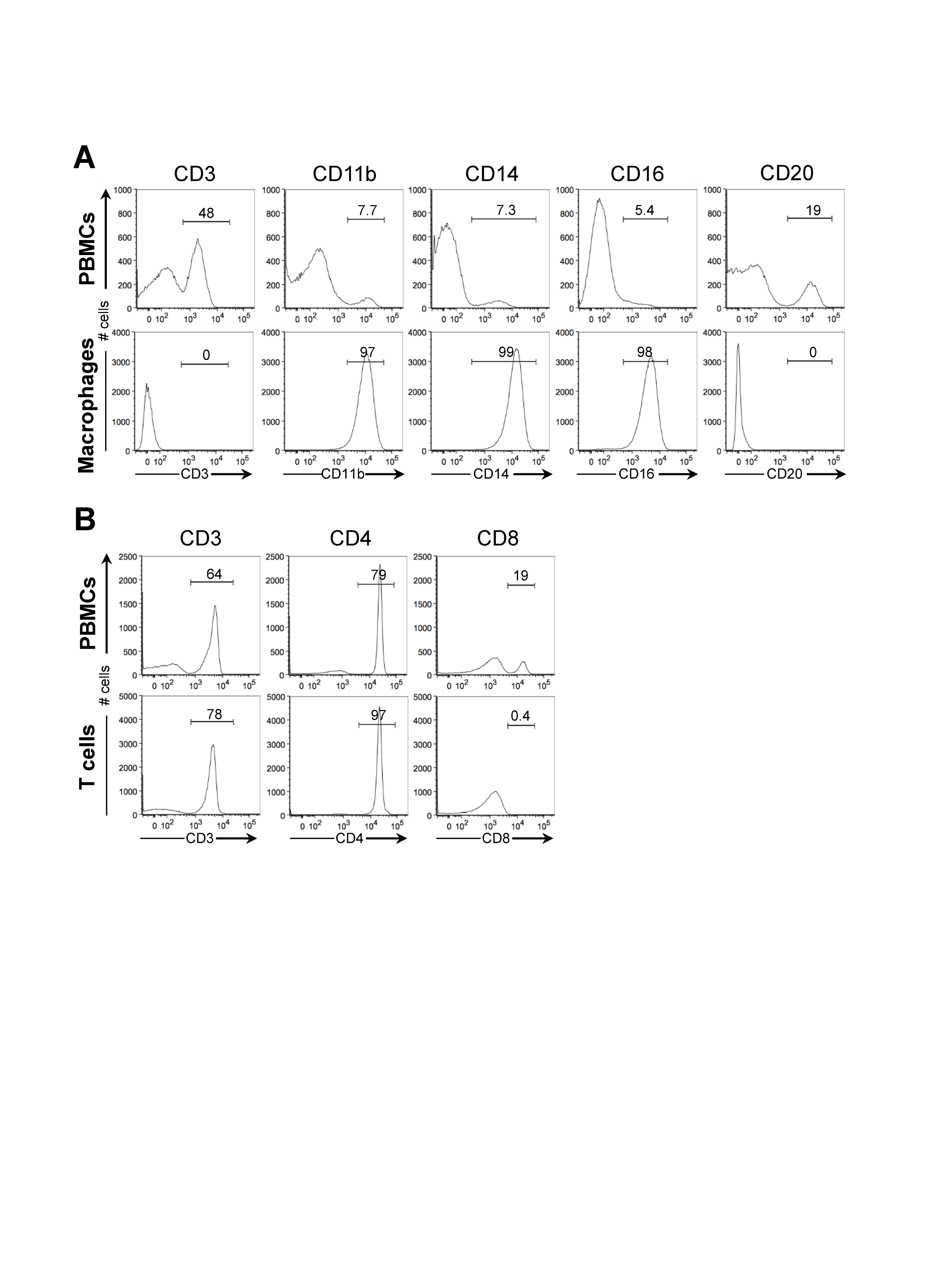

Supplement: S1 Fig — A) Rhesus macaque PBMCs and monocyte-derived macrophages were flow cytometrically stained using antibodies targeting macrophage (CD11b, CD14, CD16), T cell (CD3), and B cell (CD20) markers. Representative data from four different experiments are presented. B) Rhesus macaque PBMCs or purified CD4+ T cells were stained for flow cytometry using antibodies specific for T cells (CD3) or T cell subpopulations (CD4, CD8). Representative data from two independent experiments are shown. For A) and B), the y-axis represents the cell count, while the x-axis indicates marker signal intensity. Proportions of cells within gates are denoted above the bars. (TIF) [file ppat.1012190.s001.tif]

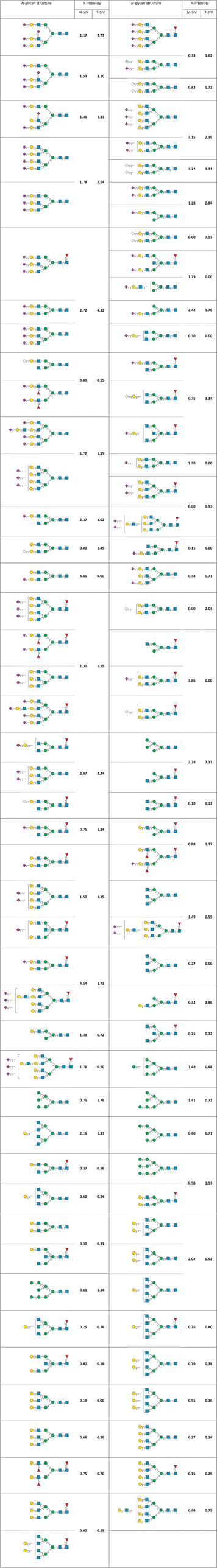

Supplement: S2 Fig — Relative peak abundances are presented as percentages of the total peak intensity (peaks 1–96 = 100%). N-glycan structures were assigned to peaks based on migration times matching the entries of an in-house N-glycan database. Symbols used to depict N-glycan structures are given in Fig 1A. (TIF) [file ppat.1012190.s002.tif]

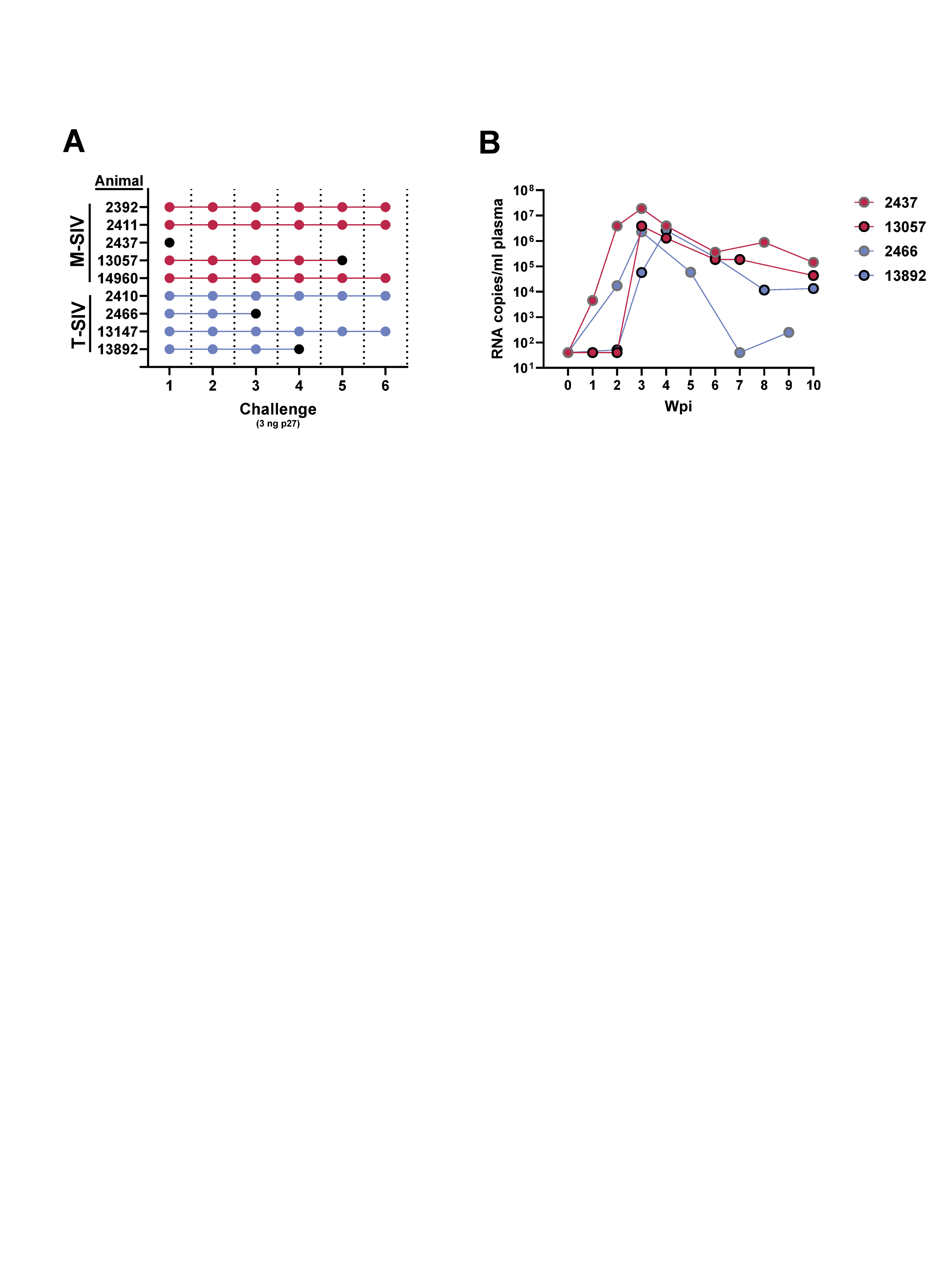

Supplement: S3 Fig — A) Rhesus macaques (n = 4–5 per group) were rectally challenged with 3 ng p27-capsid-protein of M-SIV or T-SIV diluted in PBS. The challenges were repeated every three weeks until the animals became infected (indicated by black filled symbols) or up to six challenges. Infection was determined by detection of SIV RNA in the peripheral blood by quantitative reverse transcriptase-polymerase chain reaction (RT-PCR). Animal identifiers are indicated on the y-axis. B) Plasma viral load of rhesus macaques infected with M-SIV or T-SIV was measured as RNA copies/ml from the day of challenge (day 0) up to 10 weeks post infection (wpi). At 3 wpi with SIVmac239/316 Env, animal 13057 underwent an additional challenge with SIVmac251 as part of a separate experiment, which was not part of this study. (TIF) [file ppat.1012190.s003.tif]
