# Supplementary material for: Macrophage- and CD4+ T cell-derived SIV differ in glycosylation, infectivity and neutralization sensitivity
Source: PLoS Pathog. 2024 May 28;20(5):e1012190. doi: 10.1371/journal.ppat.1012190 (PMC11161069; doi:10.1371/journal.ppat.1012190)
Supplement: Attachment — Submitted filename: 143087_1_rebuttal_2961493_s76s7p.pdf [file ppat.1012190.s004.pdf]

Full Revision

Manuscript number: RC-2024-02335
Corresponding authors: Pöhlmann, Stefan & Karsten, Christina B.

1. General Statements

We would like to express our gratitude to Reviewers 1, 2 and 3 for dedicating their time to assess this manuscript and for sharing their invaluable expertise on the subject matter. We have incorporated most of the suggestions made by reviewers but regrettably we were unable to conduct any additional experiments. This is because we chose to work with a single pooled virus stock for both $CD4^+$ T cell and macrophage-derived simian immunodeficiency virus (SIV, T-SIV, M-SIV) throughout the whole study. While this heightened our data quality by omitting donor-induced variations into our virus productions, accurately estimating the total amount of viruses needed for this project was difficult, and at this point we have depleted our reserves of M-SIV and T-SIV. Nevertheless, we are confident that our manuscript has been substantially improved in response to the reviewer's feedback, and we firmly believe that this study holds considerable implications for the development of new biomedical interventions against the human immunodeficiency virus (HIV).

Please see below in blue our point-by-point responses to the reviewer's comments and concerns. All manuscript references refer to the revised manuscript, in which the key changes have been tracked.

2. Point-by-point description of the revisions

Reviewer #1:

<u>Major comments:</u>
1 - As noted above, the differences in glycosylation are difficult to understand without more background and perhaps a figure, but it is also not clear that the changes described between lines 119 and 137 are biologically or statistically meaningful. For example, does it matter that more M-SIV virions have glycans with four antennae than T-SIV? Are there other data that show this or could experiments be done to specifically cleave these glycans at certain points to reduce their complexity and show that the infectivity differences between M-SIV and T-SIV disappear? Further, it is difficult to confirm the statement on lines 124-125 that "profiles of complex-type N-glycans differed between the two viruses (Fig. 2C)", as no statistical tests were done to compare the glycosylation being detailed in the M-SIV and T-SIV. It is more appropriate to make note that there are minor between M-SIV and T-SIV or run specific statistical tests on the data.
These are very good remarks. We have introduced a new figure (Fig. 1A) to illustrate the various glycan types, moieties, and structures discussed in the paper. Additionally, we have modified Fig. 1D-G with visualizations of the investigated *N*-glycans and adjusted and clarified

the text to enhance accessibility for experts not specializing in glycobiology (line 62-66, 204-210, 269-273). With our current dataset, we are unable to establish a direct correlation between glycosylation and functional outcomes. Consequently, we can only speculate about the potential impact of certain glycan characteristics on SIV viral functions, a field that remains largely unexplored. Furthermore, due to the absence of remaining virus samples, we are unable to conduct experiments to validate any potential direct relationship between glycosylation patterns and viral functions. It's important to note that no statistical tests were applied to the glycan analysis data, we have now emphasized this in the revised text (line 13-16, 145-149, 250-254).

2 - On line 161, the authors note that the results showing that "the virus-producing cell has a broader impact on SIV infectivity beyond its influence on Env incorporation." This is certainly one possibility that is suggested by these and prior data, there are also other possibilities. For example, the impact of Env is not linear and perhaps a certain number of Env need to be engaged, creating some kind of threshold effect that means that the virions with fewer Env just have less infectivity. Given that there is significant data that virions are generated in different locations in macrophages and T-cells, this could also be a function of which specific membrane areas in different cell types that Env embeds in, or it could be something else associated with Env that is not cell type specific.
We agree and have adapted the text to emphasize that our interpretation is just one possible interpretation, which assumes a linear relationship between Env incorporation and SIV infectivity (line 176-178).

3 - For the studies in Figure 5, looking at the direct vs. indirect infectivity, it is not clear why CEMx174 R5 cells (a T-cell/B-cell hybrid line) were used instead of primary macrophages or T-cells, or macrophage or T-cell lines or a fully agnostic cell type. This would be more convincing tested on primary cells, or at least comparing in a myeloid lineage line as well.
In this experiment, we followed a previously published protocol, which utilizes the CEMx174 R5 indicator cell line as target cell for an easy read-out of the results. We agree that the use of the suggested target cell types would be a useful extension of our existing work. Regrettably, due to the lack of remaining viruses, we are unable to fulfil this request.

4 - In Figure 6, it is not clear that VSV-G pseudotyped virus is an appropriate control, as it enters via the acidified endosome pathway and not via similar processes as the T- and M-SIV derived virions. While this may show that the glycans can bind to CBA to inhibit entry, it could also mean that the general process of endocytosis is not as susceptible to CBA inhibition and this difference in pathways should be noted as a caveat.
We acknowledge this caveat and have incorporated a statement into the results section to inform readers about the potential impact of differences in the viral uptake pathways between VSV and HIV/SIV (line 219-223).

5 - A very large number of cell lines were used, and it is not clear why experiments were done using so many different indicator or target lines, instead of performing most assays in a single

line or set of lines so that they are comparable across experiments. Some discussion of the rationale for this would be helpful.
Thank you for bringing your perspective to our attention. By selecting the specific cell lines for our experiments, we have adhered to established norms within our research domain. Specifically, 293T cells, C8166, and TZM-bl cells are standard choices for virus production via transfection, SIV titration, and infectivity assays, respectively. Moreover, in conducting the transmission assay, we followed a previously published protocol. We have revised the text to elucidate the rationale behind our selection of cell lines for different assays, omitted the potentially perplexing reference to the C8166 cell line and included references to support our cell line choices (lines 160-163, 187-188, 215-217, 458-459, 476).

Minor comments

1 - Inclusion of the p27 data characterizing the amount of virus in M-SIV and T-SIV stocks (line 95) should be shown as at least a supplemental figure or could easily be added to figure 1.
Agreed. We have included the information about the p27-concentration, and additionally the information about the infectious units and RNA copy numbers of M-SIV and T-SIV into the results section (line 98-101).

3 - The figures are relatively thin and could be combined with other figures to better connect the experiments. For instance, Figure 1 could serve as panel A for what is currently listed as Figure 2 because it is a preliminary data to the experiments in Figure 2.
As suggested by the reviewer, we have combined Fig. 1-2 (now Fig. 1), and additionally Fig. 3-5 (now Fig. 2), and 6-7 (now Fig. 3) to enhance the connections between the experiments.

3 - The authors should include quantitation of the Western blot data in Figure 1 in an adjoining graph.
We appreciate the suggestion. However, the purpose of Fig. 1B (formerly Fig. 1) was to visually represent gp120 of M-SIV and T-SIV following all treatment conditions, which produce bands of varying intensities and widths. Consequently, the bands for PNGaseF-digested gp120 appear relatively thick, which typically hampers accurate quantification. Therefore, we made the decision not to quantify the results of these blots.

4 - The legend states that the results in Figure 7 were obtained from two independent experiments (line 778), each with 3 technical replicates. As this represents only 2 biological replicates, and the experiments were performed in easily accessible cells (TZM-bl), they should be performed 1 - 3 more times to provide a more appropriate and robust data set for statistical analysis.
We agree with the reviewer that this might further improve the results but unfortunately, due to the lack of remaining pooled virus from this study, we are unable to fulfill this request.

Full Revision

Reviewer #2:

Major comments:
It appears to this assessor that some of the supplementary data can be brought to the front as part of the main figures for presentation.
We appreciate that you consider our supplementary data worthy enough to be part of the main figure set. We have now included Table S1 into the main figure set as Table 1.

CURRENT figures 3, 5, and 7 can be combined into one figure.
Similarly, CUREENT Figure 4, and 6 can also be grouped.
Alternatively, incorporating additional approaches in each set of figures to tighten the claims.
Agreed. To address this suggestion, we have combined Fig. 1-2 (now Fig. 1), 3-5 (now Fig. 2), and 6-7 (now Fig. 3).

Karsten et al pitched their story as glycosylation of SIV from different primary cells are linked to different functionality in its title and abstract, yet the authors then declared in discussion (line 318) that establishing a direct link between Env glycosylation and viral functions is technically challenging and beyond the scope of the study. This assessor feels that authors need to decide whether current manuscript should be a descriptive study (which is more fitting for a less impactful journal) or a study with further mechanistic insights.
Thank you for bringing this to our attention. We have modified the text in the introduction, results, and discussion section to underscore that our work is only suggesting but not directly proving differential glycosylation as cause for functional differences between M-SIV and T-SIV (lines 71-75, 155-157, 211-215, 251-256, 320-322).

Table S1 is highly important and should be part of the main figure. Specifically, authors took the opportunity to highlight the differential % of sialic acid terminal glycans in line 133. The charge of the sialic acids would be simple mechanism for these M-SIV particle to attach. Authors should consider some of the described nano-luciferase based viral particle attachment assays used in HIV-glycan biology. Authors should be able to treat SIV (or SIV VLPs) with sialidase to quantify the role of sialic acids on binding.
Thank you for appreciating the details of our glycan analysis. We have now included Supplementary table 1 into the main figure set as Table 1. Due to the lack of remaining virus, we are unable to address the interesting suggestions for further experiments.

As authors carefully pointed out (throughput the manuscript) that the identity (and biology) of the producer cells can have profound impacts on glycosylation events of viral particles that are being produced. This assessor was then interested to understand precisely how their simian PBMCs and monocytes derived macrophages were prepared. Additional details in M&M would be very helpful.
We realized that indeed experimental details appeared to be missing in this section since it was not obvious that kits for magnetic bead isolation have been used to isolate the cells, and adapted the text to make this more clear (395-413).

With the emphasis of cell type and glycosylation relationship, it is puzzling that authors would have chosen to use TZM-bl (artificially engineered cell line) and spinoculation (2hr to push the viruses down to cell surface with 870 x G force) in Figure 3 for comparison of M-SIV and T-SIV infectivity. To this assessor, this assay neglected the biological roles of SIV glycans. In context, 870 x G is ~150x higher than most human can withstand.

While we appreciate the reviewer's feedback, we respectfully disagree. The TZM-bl cell line has long been established as the standard cell line for SIV/HIV infectivity assays and neutralization assays in clinical trials (Sarzotti-Kelsoe et al., 2014). In this project, our initial aim was to conduct infectivity assays on a standard cell line before transitioning to more biologically relevant target cells. However, due to limited virus availability, these studies could not be completed and will be addressed in future studies.

The application of methods to increase virus-cell contact to increase cell infection of cell lines is wide-spread in HIV research. Larger virus quantities could have been used instead of spin infection, which would require the introduction of larger amounts of conditioned cell supernatant from the virus production into the experiment with potential influence on the outcomes. Another option would have been to expose the viruses to even stronger forces during ultracentrifugation to concentrate virus stocks, or to employ "sticky" reagents such as DEAE-dextran, which might generate virus aggregates (Davis et al., 2004). Considering these options, we deemed spin infection to have the smallest overall impact on our experiments, while delivering the most robust results.

Finally, we like to note that sensitivity to force between humans and cells is not comparable and cells can withstand much higher forces than humans. We kindly refer reviewer 2 to the work of Kodaka and colleagues. They carefully assessed the efficiency and impact of spin infection using retroviral constructs on primary human cells, and determined that the best conditions were 2,800xg for 90 min considering important parameter's such as cell viability, proliferation and *in vivo* differentiation.

Sarzotti-Kelsoe M, Bailer RT, Turk E, Lin CL, Bilska M, Greene KM, Gao H, Todd CA, Ozaki DA, Seaman MS, Mascola JR, Montefiori DC. 2014. Optimization and validation of the tzm-bl assay for standardized assessments of neutralizing antibodies against hiv-1. *J Immunol Methods,* 409, 131-146. DOI:10.1016/j.jim.2013.11.022.

Davis HE, Rosinski M, Morgan JR, Yarmush ML. Charged polymers modulate retrovirus transduction via membrane charge neutralization and virus aggregation. Biophys J. 2004 Feb;86(2):1234-42. doi: 10.1016/S0006-3495(04)74197-1. PMID: 14747357; PMCID: PMC1303915.

Kodaka Y, Asakura Y, Asakura A. Spin infection enables efficient gene delivery to muscle stem cells. Biotechniques. 2017 Aug 1;63(2):72-76. doi: 10.2144/000114576. PMID: 28803542; PMCID: PMC5768144.

Using a single antibody DA6 (in Figure 2, cited Edinger 2000) for Env incorporation estimation via Western seems to be crude and inadequate, even in the context of isogenic virus clone. As authors pointed out, different levels of glycosylation can affect protein folding, therefore affecting Env incorporation. By the same argument, differentially glycosylated Env protein can also impact on the ability of 'epitopes within Env protein' to be recognised by Ab. Therefore, virion incorporation of Env might not be affected, but just the detectability by a specific Ab. Western evaluation with a panel of anti-Env antibodies will help. Furthermore, quantitative proteomics coupling with glycomics would be highly useful.
We respectfully disagree on some points of your assessment. The antibody DA6 specifically targets a linear epitope in gp120 C1 (amino acids 76 to 99 in SIVmac251, Edinger et al., 2000), and the proteins analyzed by Western blot are denatured. Therefore, changes in protein folding due to differential protein glycosylation of SIV in different target cells should not affect the results. We acknowledge the potential impact of differential *N*-glycan attachment to gp120 based on the virus-producing cell on the binding of primary antibodies in Western blot analysis and agree that this issue could be mitigated by employing a panel or mixture of primary antibodies. However, please note that in Fig. 1B the input virus of M-SIV and T-SIV was normalized based on the signal received for gp120 removed of *N*-glycans (PNGase F digested). If the differential glycosylation of M-SIV and T-SIV gp120 would interfere with DA6 binding, we should observed noticeable differences between M-SIV and T-SIV in the signal of the undigested and mannose-reduced gp120 (Endo H) but this is not the case. Thus, we believe it is in this case sufficient to use only the antibody DA6 for gp120 detection.

Edinger AL, Ahuja M, Sung T, Baxter KC, Haggarty B, Doms RW, Hoxie JA. 2000. Characterization and epitope mapping of neutralizing monoclonal antibodies produced by immunization with oligomeric simian immunodeficiency virus envelope protein. *J Virol,* 74(17), 7922-7935.

It is understood that T-SIV were pooled from supernatant derived from 9 animals of PBMCs. Levels of p27 production (presumed as particles but including free p27 in reality) from each animal donor should be listed in supplement. Similar types of details should be made available for M-SIV that were derived from 8 animal donors of macrophages. qPCR estimations on the levels of viral particles production in T-SIV and M-SIV from primary cell culture amplifications appear to be already available, such information should be included in supplementary to strengthen the authors' estimated / relationships amongst glycosylation, virion Env incorporation levels, and viral particle productions are carefully controlled.
Thank you for your suggestion. We opted to include only supernatants containing more than 10 ng p27/ml in the pooled virus that constituted M-SIV and T-SIV. Consequently, we did not determine the p27 concentrations of each virus harvests below this threshold. As a result, we are unable to present replication curves for every virus production. However, we are able to provide additional information on the pooled viruses and now included the information about the p27 concentration, infectious units, and genome copy number of M-SIV and T-SIV (line 98-101).

Non-glycan biologists generally do not appreciate some of the fine details in glycan biology. The T-SIV and M-SIV system is a great model system to decode some of the functionality of glycan biology. The current team should have (in my opinion) a clear graphic representation on describing what types of different glycans in T-SIV and M-SIV are likely to contribute to the potential differences in biological outcomes. Such incorporation will guide non-glycan biologists to better appreciate the focus and the directions of authors, thereby further improving the citation of this work when it is published after peer reviewed. Importantly, focusing a specific question to be addressed may help to consolidate effort to accelerate publication of this work. A beautiful story line, just need to cross many 't' and dot a few 'i' in my view.
Thank you for sharing the excellent suggestion. We have now incorporated a new figure (Fig-1A) to help the reader with understanding the glycan biology in our manuscript. We have further adapted Fig. 1D-G to include glycan structures to provide a visual representation of the assessed *N*-glycan subgroups. Finally, we adapted the text throughout the manuscript to improve the reader's comprehension of our work (line 62-66, 204-210, 269-273).
To address your concerns regarding the clarity of the research question, we revised the text to become more specific (line 155-157, 277-283, 294-300, 317-319, 320-322, 329-338, 364-365).

Most primate centres often incorporate transcriptomic studies in their animal works. It will be helpful for the audience if the authors could provide additional transcriptomic data (with a focus on glycosylation related genes) of simian CD4+ T cells, simian macrophages, SIV infected simian CD4+ T cells, and SIV infected simian macrophages. These data will improve the comprehensiveness of this study (and should not require any major wet-lab studies) and add weight on the arguments of the authors.
This is an interesting suggestion and should be considered in future studies. Here, we choose to focus exclusively on the investigation of the viruses but not the host cell itself. Nevertheless, we discuss the existing transcriptomics data of glycosylation relevant genes in simian $CD4^+$ T cells and macrophages published by Gaskill and colleagues (line 277-287) and find that their results provide explanations for the results of our *N*-linked glycan analysis of M-SIV and T-SIV.

Gaskill PJ, Zandonatti M, Gilmartin T, Head SR, Fox HS. 2008. Macrophage-derived simian immunodeficiency virus exhibits enhanced infectivity by comparison with t-cell-derived virus. J Virol, 82(3), 1615-1621.

Reviewer #3:

Major comments

1) Line 85 "These substitutions facilitate efficient utilization of CCR5 in the absence or at very low levels of CD4 expression (Puffer et al.2002). This makes the molecular clone studied rather unique and the authors should aim to address this throughout. It does not take away from the results presented but should be addressed.

Thank you for this suggestion. We included the information about the M-tropism of our viral strain two more times into the manuscript to emphasize this information in the discussion and the limitations of the study (lines 247-249, 345-347).

2) (Figure 1) State the predicted size differences between T-SIV and M-SIV stocks with EndoH digestion (and similar for all 3 runs?).

We would have liked to address this suggestion but despite corresponding with other experts and literature research; we were unable to identify a tool, which would allow us to make such predictions.

3) Line 95 Data should be shown. This describes infectivity and could incorporated within Figure 3 along with infection of the TZM-bl cells. Infectivity of T-SIV and M-SIV on primary CD4 T-cells and macrophages is of importance. This would only be possible I assume if p27 levels were measured at each time-point collected.

We agree that the manuscript could be extended by virus replication curves on primary cells. However, we chose to include only supernatants with p27 concentrations exceeding 10 ng/ml in the pooled virus comprising M-SIV and T-SIV. Consequently, we did not determine p27-concentrations for virus harvests below this threshold in all cases, preventing us from presenting replication curves for every virus production. We have updated the results section to incorporate the total p27-concentration of M-SIV and T-SIV (lines 98-101). Prior to the submission of this manuscript, we had initiated replication assays of M-SIV and T-SIV on primary rhesus $CD4^{+}$ T cells and macrophages were initiated but these could not be completed due to the depletion of available virus.

4) There is a lack of statistical difference in the results shown in Figure 2. I assume this is due to a single measurement, but can comment be made on likelihood of biological significance with such difference between values.

Indeed, we did not conduct statistical analysis on this dataset because, given that a single pooled virus for M-SIV and T-SIV was utilized, only one measurement exists. Although we agree with the assessor on the importance of discussing the potential biological significance of the identified glycosylation differences, we are of the opinion that there is currently insufficient evidence in the literature to make scientifically grounded arguments on this matter.

5) (Figure 3) On TZM-bl cells the T-SIV stock shown 55-fold lower infectivity compared to M-SIV. This is the reverse as to what was found on macaque CD4 T-cells where T-SIV showed a 6.5-fold higher infectivity than M-SIV? Needs addressing. Again, this should be considered in

context of the results with CEMx174 R5 cells where infection between the 2 stocks appears to be similar (Figure 6).
Thank you for raising these points. We like to remark that p27-content and infectivity are not absolutely linked (Narayan et al. 2023). Virus productions also contain empty or non-functional virions and the proportions can differ depending on the virus producer cell. Thus, T-SIV having a lower infectivity per ng 27 is likely a result of cell type dependent variation in the proportion of non-functional virions and does not represent an inconsistent result.

Kedhar Narayan, Jeongpill Choi, Shreyas S. Gujar, Aidan McGraw, Hasset Tibebe, Lilia Mei Bose, Caroline N. Arnette, Taisuke Izumi, Identifying Discrepancies in HIV Infectivity and Virion Maturation Using FRET-Based Detection and Quantification, bioRxiv 2023.12.25.573317; doi: https://doi.org/10.1101/2023.12.25.573317

6) (Figure 5) The result may look cleaner if No Lectin value is subtracted from the cell lines carrying the lectin expression?
Agreed. We have adapted the figure (Fig. 2D) as suggested.

7) A much clearer introduction to CBA's would be beneficial.
We agree that this would improve our manuscript and have expanded the introduction on carbohydrate binding agents (lines 204-210).

8) A concern I have is the presentation of data in Figures 6 and 7, especially given that the cell line used is the TZM-bl cell which has been shown to be 55-fold less infectible with T-SIV. Plotting the results as % infectivity on the same graph could be somewhat misleading. Two graphs one panel for M-SIV and one for T-SIV may be easier to follow. The CEMx174 cell line may have been a better choice as similarity to infection was found? But assume those experiments were not performed?
We kindly ask reviewer 3 to note that the virus input in these experiments has been normalized for equal infectivity, as described in the figure legend. An example demonstrating the comparability of results obtained using this method can be observed in Figure 2A, right panel, which justifies our approach. While we agree that conducting neutralization assays on additional cell types might be a valuable extension of the existing work, we chose here to use the TZM-bl cell line, the standard cell line for neutralization assays in the field of HIV research (Sarzotti-Kelsoe, 2013).

Sarzotti-Kelsoe M, Bailer RT, Turk E, Lin CL, Bilska M, Greene KM, Gao H, Todd CA, Ozaki DA, Seaman MS, Mascola JR, Montefiori DC. 2014. Optimization and validation of the tzm-bl assay for standardized assessments of neutralizing antibodies against hiv-1. *J Immunol Methods,* 409, 131-146. DOI:10.1016/j.jim.2013.11.022.

9) I do feel the Discussion is extremely long and could be stream-lined to make it clearer and to the point.
To follow your valuable suggestion, we reduced the length of the discussion by approximately one-third and eliminated sections that were less directly related to our results.

10) Materials and Methods section. Is the first section on Animal studies required. Could this not just be cited if it has been previously published.
While we agree that a manuscript should be as compact as possible, we decided to include this information to ensure complete transparency regarding our animal experiments. Additionally, many journals request this information prior to publication. Consequently, we opt to retain the text in its current form.

Minor comments
1) A) needs to be removed from Figure legend 1.
Please note that we have not addressed this suggestion since Fig. 1 is now a multi-graph figure.

2) Line 160 I assume this the result from (Fig 3A).
That is correct. We have included the figure reference to make this line of text more clear.

3) Line 255-257 Difficult sentence to understand.
Thank you for making this point. This sentence has been removed in our efforts to shorten the discussion.
